# DNA Methyltransferases Are Required to Induce Heterochromatic Re-Replication in Arabidopsis

**DOI:** 10.1371/journal.pgen.1002808

**Published:** 2012-07-05

**Authors:** Hume Stroud, Christopher J. Hale, Suhua Feng, Elena Caro, Yannick Jacob, Scott D. Michaels, Steven E. Jacobsen

**Affiliations:** 1 Department of Molecular, Cell, and Developmental Biology, University of California Los Angeles, Los Angeles, California, United States of America; 2 Department of Biology, Indiana University, Bloomington, Indiana, United States of America; 3 Howard Hughes Medical Institute, University of California Los Angeles, Los Angeles, California, United States of America; National Institute of Genetics, Japan

## Abstract

The relationship between epigenetic marks on chromatin and the regulation of DNA replication is poorly understood. Mutations of the H3K27 methyltransferase genes, *ARABIDOPSIS TRITHORAX-RELATED PROTEIN5* (*ATXR5*) and *ATXR6*, result in re-replication (repeated origin firing within the same cell cycle). Here we show that mutations that reduce DNA methylation act to suppress the re-replication phenotype of *atxr5 atxr6* mutants. This suggests that DNA methylation, a mark enriched at the same heterochromatic regions that re-replicate in *atxr5/6* mutants, is required for aberrant re-replication. In contrast, RNA sequencing analyses suggest that ATXR5/6 and DNA methylation cooperatively transcriptionally silence transposable elements (TEs). Hence our results suggest a complex relationship between ATXR5/6 and DNA methylation in the regulation of DNA replication and transcription of TEs.

## Author Summary

Before cell division the genome is required to replicate once to ensure that each daughter cell inherits a full copy of genomic DNA. Eukaryotic DNA is wrapped around histones to form nucleosomes. Chemical modifications of DNA and histones are known to regulate gene expression. There is growing evidence that these modifications also regulate DNA replication, however very little is understood. Two histone methyltransferases, ARABIDOPSIS TRITHORAX-RELATED PROTEIN5 (ATXR5) and ATXR6, are required to prevent over-replication of normally silent regions of the genome called heterochromatin. Heterochromatin is enriched with transposable elements (TEs) that are silenced by modifications such as DNA methylation. We find that losses of DNA methylation suppress the over-replication defect in an *atxr5 atxr6* mutant background. This suggests that DNA methylation positively regulates DNA replication in the absence of ATXR5/6. We further study the relationship between ATXR5/6 and DNA methylation in regulating the expression of TEs and find that they cooperatively silence TEs. Together these findings reveal relationships between DNA and histone modifications in regulating basic biological processes such as DNA replication and gene expression.

## Introduction

Faithful DNA replication requires that each origin of replication fire only once per cell cycle. Re-replication has recently been suggested to be an inducer of gene copy number changes and hence threatens genome stability [1]. Multiple mechanisms that prevent re-replication are known [2], but the regulation of DNA replication at the level of chromatin remains elusive. Especially poorly understood is DNA replication of heterochromatin, which is late replicating in both plants and animals [3], [4], [5]. In Arabidopsis, heterochromatin is primarily pericentromeric and is enriched in repetitive elements such as transposons that are transcriptionally silenced by epigenetic modifications such as DNA methylation (in both CG and non-CG sequence contexts), H3 lysine 9 dimethylation (H3K9me2) and H3K27me1 [6]. The ATXR5 and ATXR6 methyltransferases catalyze H3K27 monomethylation and function redundantly to suppress over-replication of heterochromatin, likely by inhibiting re-replication [7].

Given the close correlation between sites of re-replication and sites enriched with DNA methylation, we investigated the role of DNA methylation in *atxr5 atxr6-*induced re-replication. We found that loss of DNA methylation suppressed the re-replication phenotype of *atxr5 atxr6* mutants, suggesting a role for DNA methylation in re-replication. We also profiled the transcriptome in different mutants by RNA sequencing (RNA-seq), and found that many TEs are cooperatively silenced by ATXR5/6 and DNA methylation.

## Results/Discussion

### Re-replication in *atxr5 atxr6* mutants is closely confined to heterochromatin

**Figure 1:**
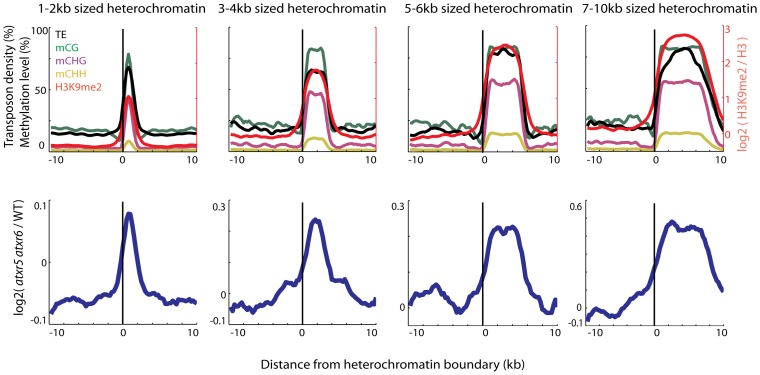
Heterochromatin is specifically re-replicated in *atxr5 atxr6* double mutants. Top panels show enrichment of DNA methylation (CG, CHG, CHH, where H = A,T or C) [11], transposable element (TE) densities (TE per base-pair) and H3K9me2 [8] over the boundaries of heterochromatic regions of indicated sizes. Values were plotted +/−10 kilobase from the boundary of heterochromatin in 500 bp bins. x = 0 is the heterochromatin boundary, x<0 is outside the region, and x>0 is into the region. Bottom panels show the distribution of DNA contents from *atxr5 atxr6* mutants relative to wild-type (log2 ratios) [7]. Heterochromatic regions were defined using previously characterized H3K9me2 regions [8]. Plots in both top and bottom panels were smoothed by taking the moving average over +/−1 bins and +/−3 bins, respectively.

We previously found that most re-replicated sites overlapped with heterochromatin [7], but the extent of the overlap has not been examined. We utilized Illumina sequencing to examine the DNA contents of *atxr5 atxr6* double mutants at the boundaries of previously defined heterochromatic patches in the arms of chromosomes [8]. Interestingly we found that DNA content in *atxr5 atxr6* decreased sharply at the boundaries of the heterochromatic patches (Figure 1). Moreover, the sizes of re-replicated regions closely tracked the sizes of defined heterochromatin (Figure 1), suggesting that re-replication is closely confined to heterochromatin and is unable to spread into flanking euchromatin. The close correlation of re-replication with heterochromatin led us to hypothesize that certain marks of heterochromatin, such as DNA methylation, may be required for the occurrence of aberrant replication.

### Loss of DNA methylation suppresses the re-replication defect in *atxr5 atxr6* mutants

To test the role of DNA methylation in re-replication, we crossed *atxr5 atxr6* mutants to *met1*, *cmt3* and *ddm1* mutants, to reduce CG, non-CG, and both CG and non-CG methylations, respectively. Previous studies indicated that *atxr5 atxr6* mutants themselves show no reduction of DNA methylation [9] and loss of DNA methylation does not affect H3K27me1 levels [10], suggesting that DNA methylation and H3K27me1 are independent of each other. To confirm that DNA methylation levels were decreased in *ddm1 atxr5 atxr6* triple mutants we performed whole genome bisulfite sequencing (BS-seq) on *atxr5 atxr6* and *ddm1 atxr5 atxr6* mutants. We observed significant loss of DNA methylation in *ddm1 atxr5 atxr6* backgrounds (Figure S1).

**Figure 2:**
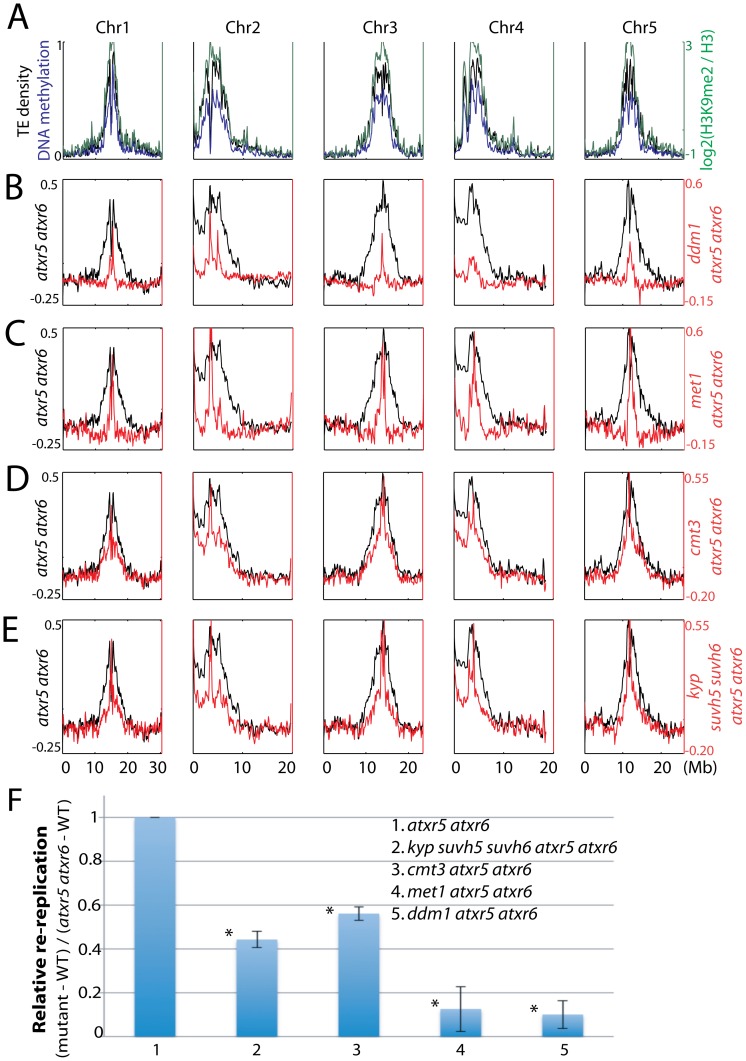
Relationship between ATXR5/6 and DNA methylation in regulating DNA replication in heterochromatin. A. Chromosomal distribution of transposable element (TE) density, DNA methylation [20] and H3K9me2 [8] data are presented to mark the locations of pericentromeric heterochromatin. B. Chromosomal views of the log2 ratio of genomic DNA reads of *atxr5 atxr6* mutants to wild type (WT) are shown in black, and the log2 ratio of *ddm1 atxr5 atxr6* mutants to WT are shown in red. C. *met1 atxr5 atxr6* mutants, D. *cmt3 atxr5 atxr6* mutants, and E. *kyp suvh5 suvh6 atxr5 atxr6* mutants. F. Quantitation of reads in heterochromatin in mutants. Fraction of reads falling into previously defined pericentromeric heterochromatin [8] was calculated. *P<10^−5^ relative to *atxr5 atxr6* mutants.

To compare genomic DNA contents between similar cell types, we sorted and collected 8C nuclei from leaves and sequenced the DNA. We chose 8C nuclei because we previously showed high levels of heterochromatin re-replication in nuclei of this ploidy level as compared to 2C or 4C nuclei (Figure S2A) [7]. Strikingly, by examining the distribution of sequenced reads across the chromosomes we observed suppression of heterochromatic re-replication in all the mutants compared to *atxr5 atxr6* mutants (Figure 2A–2D, 2F). This suggested that factors involved in DNA methylation maintenance are required for re-replication in *atxr5 atxr6* mutants. Consistently, *ddm1* and *met1*, which show the most dramatic losses of DNA methylation [11], most significantly suppressed re-replication (Figure 2B, 2C, 2F). The relatively weak but reproducible suppression in *cmt3 atxr5 atxr6* mutants could be explained by the fact that non-CG sites are relatively lowly methylated (<7%) in Arabidopsis [11].

We next tested the role of H3K9me2 in regulating re-replication by generating *kyp suvh5 suvh6 atxr5 atxr6* quintuple mutants. Because *kyp suvh5 suvh6* mutants are depleted in both H3K9me2 and non-CG methylation, we reasoned that if H3K9me2 played a dominant role in regulating re-replication, *kyp suvh5 suvh6 atxr5 atxr6* mutants should exhibit a stronger degree of suppression of re-replication than do *cmt3 atxr5 atxr6* mutants, which lose non-CG methylation but retain a significant amount of H3K9me2 [12]. However, we observed a very similar degree of suppression of re-replication in *kyp suvh5 suvh6 atxr5 atxr6* mutants compared to *cmt3 atxr5 atxr6* mutants (Figure 2E, 2F; Figure S3). Hence the reduction of DNA re-replication observed in *kyp suvh5 suvh6 atxr5 atxr6* mutants is likely due to losses of DNA methylation rather than losses of H3K9me2. We also crossed *atxr5 atxr6* to *drm1 drm2* double mutants. DRM1 and DRM2 maintain asymmetric methylation at a subset of cytosines. Consistent with the fact that *drm1 drm2* mutants show only limited reductions in DNA methylation genome-wide [11], we did not observe significant suppression in *drm1 drm2 atxr5 atxr6* quadruple mutants (Figure S4).

Finally, we crossed *atxr5 atxr6* to *mom1*, which exhibits transcriptional derepression of TEs without altering DNA methylation [13]. We did not observe significant suppression of re-replication in *mom1 atxr5 atxr6* mutants (Figure S5), further supporting our hypothesis that it is the loss of DNA methylation that causes suppression of re-replication. Flow cytometry analyses on multiple biological replicates of all the mutants confirmed the results obtained by DNA sequencing (Figure S2B). It should be noted that DNA methylation single mutants in wild type ATXR5 ATXR6 backgrounds did not cause significant changes in DNA content (Figure S2C). In sum, these results suggest that DNA methylation plays a role in the induction of re-replication in *atxr5 atxr6*, consistent with the hypothesis that DNA methylation promotes DNA replication in heterochromatin.

### Relationship between transcriptional derepression and re-replication in *atxr5 atxr6* mutants

**Figure 3:**
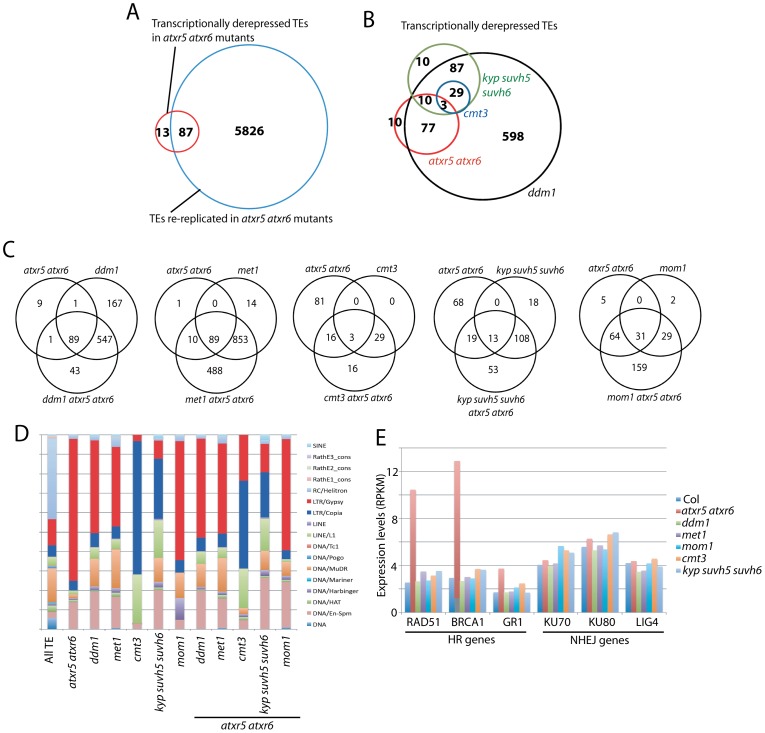
Relationship between ATXR5/6 and DNA methylation in transcriptionally silencing TEs. A. Overlap of re-replicated TEs in *atxr5 atxr6* mutants, with transcriptionally reactivated TEs in *atxr5 atxr6* mutants. B. Overlap of TEs transcriptionally derepressed in *atxr5 atxr6* mutants and DNA methylation mutants. C. Overlap of TEs derepressed in indicated mutants. D. TE families of derepressed TEs in indicated mutants. E. Normalized expression levels of key genes in homologous recombination (HR) and non-homologous end-joining (NHEJ) DNA repair pathways.

Analyses at a few loci have shown that *atxr5 atxr6* mutants exhibit transcriptional derepression of certain TEs [9]. A possible explanation for the TE reactivation could be that more permissive chromatin assembled on additional DNA copies of TEs resulting from re-replication might allow for transcription to occur. Conversely, it is also possible that the transcriptional derepression in *atxr5 atxr6* mutants may in some way be causing the re-replication defect. To examine the relationship between heterochromatin re-replication and transposon derepression in *atxr5 atxr6* mutants, we analyzed the transcriptome of *atxr5 atxr6* mutants by performing RNA-seq on cotyledons, which we found show significant re-replication (Figure S6). We defined 100 TEs that were consistently upregulated in biological replicates by applying stringent thresholds (see Materials and Methods) (Table S1). These TEs were highly methylated in wild type, and did not lose DNA methylation in *atxr5 atxr6* (Figure S7). In addition we sequenced genomic DNA from cotyledons of *atxr5 atxr6* mutants, and defined re-replicated TEs (see Materials and Methods). Importantly, a subset of TEs that were transcriptionally derepressed was not re-replicated (Figure 3A, Figure S8). The presence of TEs derepressed in *atxr5 atxr6* mutants that were not overlapping with re-replicated regions suggests that TE reactivation is not due to increased DNA copy numbers at these loci. Re-replicated TEs were much more numerous than those transcriptionally derepressed in *atxr5 atxr6* mutants, where only 1.4% of TEs that showed re-replication also showed derepression. These results suggest that heterochromatin re-replication and transposon derepression are likely two separate phenomena in *atxr5 atxr6* mutants, which is consistent with the observations that DNA methyltransferase mutants do not act as enhancers of the DNA replication defects in *atxr5 atxr6* mutants, as would be expected if there were a simple relationship between transcriptional derepression and DNA replication.

### Comparison of TEs regulated by ATXR5/6 and DNA methylation

We next examined the relationship between TEs derepressed in *atxr5 atxr6* mutants and DNA methylation mutants. Again, TEs were defined to be derepressed using stringent thresholds. We found significant overlap between TEs derepressed in *ddm1* and *met1* mutants (Figure S9A). And consistent with the dependence of non-CG methylation on H3K9 methylation, we found that all TEs derepressed in *cmt3* mutants overlapped with those derepressed in *kyp suvh5 suvh6* mutants (Figure 3B). Most TEs derepressed in *atxr5 atxr6* mutants overlapped with those derepressed in *ddm1* and *met1* mutants (Figure 3B, Figures S9B and S10), however there was little overlap with those derepressed in *cmt3* and *kyp suvh5 suvh6* mutants (Figure 3B). Hence ATXR5/6 and H3K9me2/non-CG methylation generally regulate different TEs at a transcriptional level.

TEs regulated by ATXR5/6 were over-represented by LTR/Gypsy type TEs, whereas TEs regulated by KYP SUVH5 SUVH6 and CMT3 were over-represented by LTR/Copia and LINE/L1 type TEs (Figure 3D). This was not necessarily predicted based on methylation levels since it was not the case that certain types of TEs were preferentially CG or non-CG methylated (Figure S11). Hence different silencing pathways tend to regulate specific types of TEs. Notably, a large subset of TEs became reactivated only when combining *atxr5 atxr6* mutants with DNA methylation mutants (Figure 3C, Figure S9D). Hence, a relatively large proportion of TEs are cooperatively silenced by ATXR5/6 and DNA methylation. It is not clear why *ddm1 atxr5 atxr6* mutants did not show as many additional TEs derepressed as *met1 atxr5 atxr6* mutants. We also found that the combination of *mom1* with *atxr5 atxr6* mutants caused activation of many additional TEs (Figure 3C and Figure S9C).

### Over-expression of DNA repair genes in *atxr5 atxr6* mutants

An additional insight from the transcriptome of *atxr5 atxr6* mutants was that genes in the homologous recombination (HR) DNA repair pathway were over-expressed (Figure 3E). This was confirmed by quantitative RT-PCR analyses (Figure S12). None of the other tested mutants showed this effect (Figure 3E), suggesting that induction of these HR genes is likely due to re-replication. Furthermore, the *ddm1* mutant which caused the most significant suppression of re-replication in the *atxr5 atxr6* background, also caused a significant suppression of the expression of these HR genes (Figure S13), further supporting the hypothesis that these HR genes are up-regulated in response to DNA damage caused by re-replication.

In summary, our results show that DNA methylation is required for the heterochromatic re-replication defect in *atxr5 atxr6*, suggesting that DNA methylation positively regulates DNA replication in heterochromatin. We further find that DNA methylation and ATXR5/6 act synergistically in the transcriptional suppression of transposons. The molecular mechanisms causing these relationships are unclear, and it may be that losing DNA methylation causes mobilization of unknown pathways that result in suppression of re-replication. Our findings suggest a complex interplay between epigenetic marks in regulating DNA replication and transcriptional silencing in heterochromatin.

## Materials and Methods

### Plant material

All mutant lines in this study were in the Columbia background. Previously characterized mutant alleles were used for crosses: *cmt3–11*, *ddm1–2*, *met1–3*, *mom1–2*, *atxr5 atxr6* [9] and *kyp suvh5 suvh6* [14]. *met1–3* mutants in both wild-type and *atxr5 atxr6* mutant backgrounds used in this study were second generation homozygous lines. *ddm1–2* mutants in both wild-type and *atxr5 atxr6* mutant backgrounds were sixth generation homozygous lines. Plants were grown under continuous light.

### Flow cytometry

For sorting nuclei: One gram of mature rosette leaves were collected from 3–4-week-old plants, chopped in 0.5 ml of filtered Galbraith buffer, and stained with propidium iodide. A BD FACS Aria II in the UCLA Jonsson Comprehensive Cancer Center (JCCC) Flow Cytometry Core Facility was used to sort the nuclei. For sequencing, 7,000–9,000 8C nuclei of each sample were collected, and purified DNA with Picopure purification kit (Arcturus) following manufacturer instructions.

For generating flow cytometry profiles: Three mature rosette leaves were pooled from separate 4-week-old plants, chopped in 2 ml of filtered Galbraith buffer, and stained with a solution of propidium iodide and RNAse A. A BD FACScan flow cytometer in the UCLA JCCC Flow Cytometry Core Facility was used to generate the FACS profiles. For each sample at least 10,000 nuclei were analyzed, and widths of peaks (coefficient of variation values) [7] were calculated using Cyflogic analysis software (http://www.cyflogic.com).

### Illumina genomic library preparation

Genomic DNA was sonicated to 200 bp with a Covaris S2, and Illumina libraries were generated following manufacturer instructions. The libraries were sequenced using Illumina Genome Analyzer II following manufacturer instructions.

### Illumina mRNA–seq library preparation

RNA–seq experiments were performed in two biological replicates for each genotype. 0.1 g of tissue was ground in Trizol. Total RNA were treated with DNaseI (Roche), and cleaned up with phenol-chlorophorm and precipitated with ethanol. Libraries were generated and sequenced following manufacturer instructions (Illumina). For verification experiments, the same RNA extraction protocol was used. Single stranded cDNA was synthesized using polyA primers and Superscript II (Invitrogen). For quantitative PCR analysis, cDNA were amplified with iQ SYBR Green Supermix (Biorad) using primers previously described [15] and *ACTIN* gene was used as an internal control. Primers are listed in Table S2.

### Whole-genome bisulfite sequencing (BS-seq) library generation

0.5∼1 ug of genomic DNA was used to generate BS-seq libraries. Libraries were generated as previously described [11].

### Illumina read alignment and analysis

Genomic DNA sequenced reads were base-called using the standard Illumina software. We used Bowtie [16] to uniquely align the reads to the *Arabidopsis thaliana* genome (TAIR8), allowing up to 2 mismatches. For all genomic libraries, reads mapping to identical positions were collapsed into single reads.

Method for defining re-replicated regions: Genome was tiled into 100 bp bins, and scores of reads for each bin were computed before the log2 ratio of *atxr5 atxr6* to wild-type was taken. Score = (# reads+c)/0.1 kb/(# million mapping reads), where c is a pseudocount defined by (# million mapping reads)/10. In this case, c_WT_ = 6.8 and c*_atxr5atxr6_* = 5.3. Pseudocounts were used to avoid divisions by zero. Next, the genome was tiled into 1 kb bins (500 bp overlap), and Z-scores of log2(*atxr5 atxr6*/wild-type) were computed. A Z>2 cutoff was applied and regions within 500 bp were merged. Transposable elements (TEs) overlapping with these regions by 1 bp were considered to be re-replicating in *atxr5 atxr6* double mutants.

Both gene and TE expression in the RNA-seq data was measured by calculating reads per kilobase per million mapped reads (RPKM) [17]. P-values to detect differential expression were calculated by Fisher's exact test and Benjamini-Hochberg corrected [18] for multiple testing. TEs upregulated in wild-type and mutants were defined by mutant/wild-type>4 and P<0.01. To avoid divisions by zero, TEs with expression levels of zero were assigned the lowest non-zero TE expression value in each sample. Only TEs defined as upregulated in all biological replicates were considered as being derepressed. TEs defined to be upregulated are listed in Table S1.

BS-seq data was mapped to the TAIR8 genome by BS Seeker [19] by allowing up to 2 mismatches. Methylation levels were computed by calculating #C/(#C+#T).

All sequencing data have been deposited at Gene Expression Omnibus (GEO) (accession number GSE38286).

## Supporting Information

Figure S1 Genome-wide comparison of losses of DNA methylation in *ddm1* mutants in wild-type and *atxr5 atxr6* backgrounds. Whole genome bisulfite sequencing (BS-seq) was used to calculate DNA methylation levels in different mutants. DNA methylation levels across all five chromosomes, as well as average DNA methylation levels over TEs and genes were plotted. Plots were smoothed triangularly (bin*_i_* = 0.25×bin*_i−1_*+0.5×bin*_i_*+0.25×bin*_i+1_*) once for chromosomal views.
(EPS)

Figure S2 Flow cytometry profiles of different mutants. A. Examples of flow cytometry profiles. B. Flow cytometry profiles of different mutants. To quantify re-replication, coefficient of variation (CV) values [7] of peaks defined by fluoresence intensity of 8C nuclei (i.e. the widths of peaks) were calculated. Data represented as mean ± SD for triplicates. C. CV values for DNA methylation mutants in absence of *atxr5 atxr6* mutations.
(EPS)

Figure S3 Chromosomal view comparison between *kyp suvh5 suvh6 atxr5 atxr6* quintuple mutants and *cmt3 atxr5 atxr6* triple mutants. The normalized density of reads (reads/base/million uniquely mapping reads) were calculated in 100 kilobase bins, and smoothed triangularly ten times in order to superimpose the two profiles.
(EPS)

Figure S4 Mutations in DRM1 DRM2 do not suppress re-replication. Genomic DNA content was measured by FACS (%CV). CV values were normalized to wild type.
(EPS)

Figure S5 Mutation in MOM1 does not suppress re-replication. Genomic DNA content was measured by FACS (%CV). CV values were normalized to wild type.
(EPS)

Figure S6 Cotyledons are re-replicated in *atxr5 atxr6*. Chromosomal views of genomic DNA sequencing reads in *atxr5 atxr6* vs WT.
(EPS)

Figure S7 DNA methylation over TEs derepressed in *atxr5 atxr6* double mutants. Average DNA methylation levels (measured by BS-seq) over all TEs and TEs upregulated in *atxr5 atxr6* double mutants. Plots were smoothed triangularly three times. TSS = transcription start sites, TTS = transcription termination sites.
(EPS)

Figure S8 DNA contents in TEs defined to be transcriptionally derepressed but not re-replicated in *atxr5 atxr6* mutants. Cotyledon genomic DNA reads per kilobase TE length per million mapping reads in WT and *atxr5 atxr6* were calculated for the 13 non-re-replicating TEs indicated in Figure 3A. Significance was assessed by Wilcoxon ranksum test.
(EPS)

Figure S9 Overlap of TEs derepressed in different mutants.
(EPS)

Figure S10 Genome-browser view examples of TE derepression. Normalized expression values (RPKM) were calculated in 20 bp non-overlapping bins. TEs are also shown.
(EPS)

Figure S11 DNA methylation over different TE families. Average wild-type DNA methylation levels (measured by BS-seq) over indicated classes of TEs. Plots were smoothed triangularly three times.
(EPS)

Figure S12 Quantitative RT–PCR analyses on homologous recombination repair genes. The values were normalized to *ACTIN* gene (Q = 2∧−ΔΔCt). Error bars represent the standard deviation.
(EPS)

Figure S13 Suppression of over-expression of HR genes in *ddm1 atxr5 atxr6* mutants.
(EPS)

Table S1 TEs defined to be upregulated in each mutant.
(XLS)

Table S2 Primers used for quantitative PCR experiments.
(XLS)
